# Regulatory T_R3-56_ Cells in the Complex Panorama of Immune Activation and Regulation

**DOI:** 10.3390/cells12242841

**Published:** 2023-12-15

**Authors:** Flavia Carriero, Valentina Rubino, Stefania Leone, Rosangela Montanaro, Vincenzo Brancaleone, Giuseppina Ruggiero, Giuseppe Terrazzano

**Affiliations:** 1 Department of Sciences, University of Basilicata, 85100 Potenza, Italy; flavia.carriero@unibas.it (F.C.); rosangela.montanaro@unibas.it (R.M.); vincenzo.brancaleone@unibas.it (V.B.); 2 Department of Translational Medicine, University of Naples Federico II, 80131 Naples, Italy; valentina.rubino@unina.it (V.R.); giuseppina.ruggiero@unina.it (G.R.); 3 Hematopoietic Stem Cell Transplantation Unit, Azienda Ospedaliera A. Cardarelli, 80131 Naples, Italy; stefania.leone@aocardarelli.it

**Keywords:** immune regulation, immune regulatory cell phenotypes, T_R3-56_

## Abstract

The interplay between immune activation and immune regulation is a fundamental aspect of the functional harmony of the immune system. This delicate balance is essential to triggering correct and effective immune responses against pathogens while preventing excessive inflammation and the immunopathogenic mechanisms of autoimmunity. The knowledge of all the mechanisms involved in immune regulation is not yet definitive, and, probably, the overall picture is much broader than what has been described in the scientific literature so far. Given the plasticity of the immune system and the diversity of organisms, it is highly probable that numerous other cells and molecules are still to be ascribed to the immune regulation process. Here, we report a general overview of how immune activation and regulation interact, based on the involvement of molecules and cells specifically dedicated to these processes. In addition, we discuss the role of T_R3-56_ lymphocytes as a new cellular candidate in the immune regulation landscape.

## 1. Introduction

The immune system protects the body from infections and maintains overall health [1,2,3]. This protection occurs through the activation of the immune system, which represents a complex process by which the immune response is stimulated in response to the presence of pathogens (such as viruses, bacteria, fungi, etc.), foreign substances, or abnormal cells (e.g., cancer cells) in the body [1,2,3,4]. This process is usually called “immune activation” and can involve various cells and immune molecules harmonically acting to create a defense against these threats [1,2,3,4,5].

Immune activation processes are expressed by the innate and adaptive immune responses of the immune system, each with distinct roles and mechanisms to defend the body against infections and other threats [1,2,3,4,5].

The innate immune response is rapidly acting and represents the first line of defense, thus providing immediate but relatively non-specific protection [1,2,3,4,5,6]. In fact, the innate response does not discriminate among specific pathogens but recognizes common characteristics shared by many pathogens, such as certain molecules on the surface of the pathogen [7,8,9,10,11].

Components of the innate immune system include physical barriers such as the skin and mucous membranes, as well as cellular and biochemical elements such as phagocytes (white blood cells that engulf and digest pathogens) [7,8,9,10,11] and natural killer (NK) cells (infected host or anomalous cells) [12,13,14].

The innate response often promotes pro-inflammatory phases and is itself triggered by inflammation, which helps in the recruitment of immune cells to the site of infection and improves the body’s overall defense [15,16,17].

The adaptive immune response develops more slowly but is highly specific and targets particular pathogens precisely, adapting to the microenvironmental conditions during the immune response itself [1,2,3]. Such a response is characterized by “immunological memory”, which is a fundamental feature of the adaptive immune system offering an effector advantage upon subsequent encounters with the pathogen [1,2,3,18,19]. In this regard, after encountering a specific pathogen, the adaptive immune system “remembers” previous encounters and responds more effectively upon subsequent exposures to the same pathogen [1,2,3,18,19].

The adaptive response involves specialized white blood cells called B and T lymphocytes [1,2,3]. Briefly, B cells are immune cells specialized in the production and secretion of antibodies, proteins specifically capable of recognizing and binding to specific antigens, expressed by pathogens such as bacteria or viruses [1,2,3,20,21]. This binding can either directly neutralize pathogens or flag them for elimination by other immune cells, thereby contributing to the battle against infections and the maintenance of immune balance in the body [1,2,3,20,21]. Moreover, T cells perform various functions [1,2,3,22], including assisting B cell functions (T helper, Th) [1,2,3,23], directly killing infected cells (cytotoxic T lymphocytes, CTL) [1,2,3,24], and regulating the immune response [25]. The functions of T cells are expressed in a marked versatility (or plasticity), which takes on considerable value in coordinating immune responses, adapting to different challenges, and guaranteeing an effective but controlled defense against infections and other threats linked to the control exercised by the immune system [1,2,3,22,23,24,25].

In a perspective, immune cells have several highly specialized roles in the body, including the identification and neutralization of threats (the effector functions of immune activation), as well as the ability to activate or inhibit the response itself (the regulatory mechanisms) [1,2,3,15,16,17,18,19,20,21,22,23,24,25,26].

## 2. The Immune Regulation

The orchestration of the immune response is a sophisticated and intricately managed process that guarantees the immune system’s efficiency while preventing exaggerated or detrimental reactions [25,26,27,28,29,30]. Regulation involves an ample network of immune cells, signaling molecules, and regulatory mechanisms that work together to maintain immune balance and prevent immune-related diseases [25,26,27,28,29,30].

In this regard, both the inappropriate initiation and incorrect termination of the immune response can lead to various serious health issues, including chronic conditions, autoimmune diseases, and even cancer [31,32,33,34,35].

On the basis of the “danger model”, originally postulated by P. Matzinger, the initiation of immune cell responses when there is no actual threat or presence of harmful pathogens (such as viruses, bacteria, fungi, etc.) in the body represents a risk for the emergence of several immune-mediated diseases [36,37,38]. Physiologically, once the immune system has eliminated the pathogen, it should return to its basal state without expressing functional residues that are dangerous for the health of the host organism’s own components [1,2,3,4,5,6,7,8,9]. The continuation of an active immune response in the absence of a threat can seriously damage the molecular and cellular components of body tissues (the self) [1,2,3,4,5,6,7,8,9].

The inappropriate initiation and non-termination of immune effector functions, dependent on an immune regulatory failure, represents the basis for immune cells to act in an autoaggressive way in the absence of the pathogen, generating damage to healthy tissues [38,39,40,41].

### 2.1. The Interplay between Immune Activation and Regulation

Taking a broader perspective on the functions and organization of the immune system, the prevailing hypothesis suggests that immune responses are remarkably flexible and adaptable [42,43,44]. Individual immune cells therefore possess the ability to adapt their functional capabilities over time, responding to the specific demands of their microenvironment, whether it is to trigger an active response (the effector or activation phase) [1,2,3,4,5,6,7,8,9,10,11,12,13,14,15,16,17,18,19,20,21,22,23,24] or to maintain control through immune regulation [25,26,27,28,29,30].

This intricate balancing act within the immune response evokes the concept of “immune plasticity” [45,46]. Consequently, it is reasonable to consider that disruptions in immune plasticity could serve as a major factor in the failure of both immune activation and regulation, resulting in immune system-related disorders such as immunodeficiencies [47] and autoimmune diseases [38,39,40,41].

Current research is dedicated to gaining insight into the mechanisms governing immune regulation and exploring new therapies tailored to address conditions related to the immune system [48].

### 2.2. The Main Features of Immune Regulation: Aspects, Molecules, and Cells

The regulation of immune responses is a complex and finely orchestrated process that involves several aspects and key mechanisms crucial to maintaining the delicate balance between an effective defense and the restoration of the state of health, avoiding harmful excessive reactions [25,26,27,28,29,30,31,32,33,34,35,36,37,38,39,40,41,42,43,44,45,46,47,48].

A peculiar feature of the immune system is the ability to distinguish between the body’s own cells and tissues (self) and foreign invaders (non-self) [1,2,3,4,5,6,7,8,9]. Discrimination between self and non-self is critical to prevent the immune system from mistakenly attacking the body cells, which can lead to autoimmune diseases [31,32,33]. Self-recognition [42,43,44] is largely based on tolerance mechanisms [42,43,44,49].

The immune system has mechanisms to recognize and tolerate self-antigens, preventing the immune response from targeting and attacking the body’s own cells and tissues [49]. Central tolerance occurs during the development of immune cells in the thymus (for T cells) and bone marrow (for B cells), where self-reactive cells are eliminated or rendered non-functional [30,49,50,51,52,53,54]. Peripheral tolerance mechanisms further suppress or regulate self-reactive immune cells in the periphery to prevent autoimmune reactions in tissue [49,55].

Cytokines are signaling molecules produced by immune cells that regulate the immune response [56]. They can have pro-inflammatory or anti-inflammatory properties. For example, pro-inflammatory cytokines like interleukin (IL)-1, IL-6, interferon-gamma (IFN-γ), and tumor necrosis factor-alpha (TNF-α) promote inflammation and immune activation [56], while anti-inflammatory cytokines like IL-10 and transforming growth factor beta (TGF-β) dampen immune responses and promote tolerance [57].

Checkpoint molecules, such as programmed cell death protein 1 (PD-1) [58] and cytotoxic T-lymphocyte-associated protein 4 (CTLA-4) [59], are involved in regulating immune responses and preventing excessive immune activation [60]. They act as “brakes” on immune cells and can inhibit their activation and effector functions [60]. Targeting these checkpoint molecules has been successful in immunotherapy approaches, particularly in autoimmunity and cancer treatment [60].

The immune system employs feedback mechanisms to regulate its own activity. Various immune cells and molecules can produce inhibitory or activating signals that modulate the immune response [23,24,25,26,27,28,29,30,56,57,58,59,60]. These feedback mechanisms help maintain immune balance and prevent excessive or prolonged immune activation [44,45,46].

The local tissue environment can profoundly influence immune responses. The presence of specific molecules or cells in tissue can boost or dampen immune reactions [8,9,10,11,36,37,38,41,42,43,44].

All these aspects can account for the enormous value of environmental factors in determining immune plasticity and, therefore, positively or negatively influencing immune regulation [42,43,44,45,46].

The scientific literature has highlighted the role of numerous cells with regulatory functions of the immune response. In this sense, the aforementioned characteristics of immune plasticity make it highly probable that immune regulation is mediated by a large and non-definitive number of cells functionally capable of being involved in immune regulation [42,43,44,45,46,47,48,49,50,51,52,53,54,55,56,57,58,59,60].

In this review, we will address the synthetic description of the main cells described as possessing immune regulation ability.

Regulatory T cells (Tregs) are a specialized subset of CD4+ T lymphocytes (T cells) that play a crucial role in immune regulation and maintaining immune tolerance [26,29,30,55,61,62]. They are essential in preventing excessive immune responses and controlling immune-related diseases, including autoimmune disorders, allergies, and graft rejection in transplantation [61,62,63]. Tregs are characterized by the expression of a transcription factor called FoxP3 (Forkhead box P3), which is considered a master regulator of their development and function [62,63]. Mutations or deficiencies in FoxP3 lead to severe autoimmune diseases [61,62,63], highlighting the critical role of Tregs in immune homeostasis.

Two subtypes of Tregs have been described: the natural constitutive (nTreg) [29,61,62,63] and the inducible (iTreg) cells [61,62,63,64]. nTregs develop in the thymus and derive from some progenitor T cells that undergo a selection process conferring them regulatory properties [29,61,62,63]. nTregs are characterized by specific surface markers, such as CD4 and CD25 (interleukin-2 receptor alpha chain) [29,61,62,63]. They have a natural ability to suppress the activation and proliferation of other immune cells, including effector T cells, which helps maintain immune homeostasis and prevent autoimmune reactions [29,61,62,63]. iTregs are generated in peripheral tissues, such as the gut or sites of inflammation, in response to specific environmental cues [61,62,63,64]. The iTreg subtype arises from the differentiation of conventional CD4+ T cells (non-regulatory T cells) in response to signals from the local tissue microenvironment and the presence of certain cytokines, such as transforming growth factor-beta (TGF-β) [61,62,63,64]. iTregs can tailor their regulatory functions to specific tissues [61,62,63,64].

Tregs use various mechanisms to suppress immune responses: They secrete immunosuppressive cytokines like interleukin-10 (IL-10) and transforming growth factor-beta (TGF-β). These cytokines can suppress the activity and proliferation of other immune cells, such as T cells, B cells, and antigen-presenting cells, thereby limiting immune activation [29,61,62,63,64]; Tregs can directly interact with and suppress the function of other immune cells through cell-to-cell contact [29,61,62,63,64]. This interaction involves molecules such as cytotoxic T-lymphocyte-associated protein 4 (CTLA-4) and lymphocyte activation gene 3 (LAG-3) on the surface of Tregs, which interact with ligands on target cells, leading to the inhibition of immune responses [63,64,65]; Tregs can also modulate the metabolic environment to suppress immune responses. They use metabolic pathways, such as increased adenosine production or the consumption of IL-2, to create an immunosuppressive milieu that dampens immune activation [61].

Tregs are crucial for maintaining self-tolerance and preventing autoimmune diseases [29,61,62,63,64,65]. They recognize self-antigens and suppress the activation and function of autoreactive T cells that could potentially cause harm to the body’s own tissues [29,61,62,63,64,65]. However, the balance between Tregs and effector T cells can be disrupted in certain conditions, leading to immune dysregulation [29,41,42,44,55,61,62,63,64,65]. The deficiency or dysfunction of Tregs can result in uncontrolled immune activation and the development of autoimmune diseases [29,41,42,44,55,61,62,63,64,65]. On the other hand, an excessive or overactive Treg response can contribute to immune suppression and hinder effective immune responses against infections or cancer [29,41,42,44,55,61,62,63,64,65].

Research on Tregs and their role in immune regulation is a rapidly evolving field. Several approaches to harnessing the therapeutic potential of Tregs in treating autoimmune diseases, allergies, transplant rejection, and other immune-related disorders have been evaluated [66,67]. Strategies include Treg-based cellular therapies and the modulation of Treg function and stability for therapeutic interventions [66,67].

CD8+ suppressor T cells represent a subtype of Tregs and have been described as having a unique ability to suppress immune responses, which may be useful in preventing autoimmune reactions [68,69,70]. CD8+ Tregs appear to be a specialized subset of cytotoxic T cells, whose functions and mechanisms of action are still not entirely clear but play several crucial roles in immune regulation [68,69,70].

In the context of T lymphocytes with immunoregulatory abilities, Type 1 (Tr1) and Type 2 (Tr2) regulatory T cells are certainly worth mentioning. The Tr1 subset plays a crucial role in regulating the immune response and maintaining immune tolerance [71]. The mechanisms and functions of Tr1 cells are not fully understood [71,72,73]. However, the scientific literature has described that Tr1 cells predominantly produce the anti-inflammatory cytokines IL-10 [71,72,73]. Such cytokines suppress the activity of other immune cells, including T cells and macrophages, dampening inflammation [71,72,73]. The ability of Tr1 cells to regulate immune responses makes them an interesting target for potential therapeutic interventions in conditions involving immune dysregulation, such as autoimmune diseases and allergies [72,73]. In this regard, it is worth noting that Tr1 cells have been described as contributing to the immune evasion of tumors by suppressing the anti-tumor immune response [72,73]. This can be a promising challenge in cancer immunotherapy. Moreover, Tr1 cells are involved in preventing excessive allergic responses by inhibiting the activation of immune cells responsible for allergy-related inflammation [73,74]. The Tr2 subset has also been described as Th3 cells and is involved in immune regulation and suppressing inflammatory responses [75,76]. They play a crucial role in maintaining immune homeostasis by dampening excessive immune activation and preventing immune-mediated tissue damage [75,76]. Tr2 cells exert their immunosuppressive effects through the secretion of TGF-β, which has anti-inflammatory properties and can inhibit the activity of various immune cells, including T cells, B cells, and APCs [75,76]. Tr2 cells have been implicated in the regulation of immune responses in a variety of contexts, including allergic reactions, autoimmune diseases, and tissue inflammation [75,76]. The differentiation and development of Tr1 and Tr2 cells are influenced by various factors, including the cytokine environment and interactions with other immune cells [77]. They can arise from different sources, including conventional CD4+ T cells that have been exposed to specific signals, as well as from the conversion of other regulatory T cell subsets [77].

Natural killer T (NKT) cells are a unique subset of immune cells that possess both T cell and natural killer cell characteristics [78,79]. These cells express both the T cell receptor (the CD3 molecule) and the natural killer cell marker (the CD56 molecule) on their surface [78,79]. NKT cells play a critical role in the immune response by bridging the innate and adaptive immune systems [80]. They recognize a variety of lipid and glycolipid antigens presented by the non-classical major histocompatibility complex (MHC) molecule, CD1d [80,81]. Upon activation, NKT cells rapidly produce large amounts of cytokines, such as IFN-γ and IL-4, which can modulate the immune response and suppress the activation and proliferation of other immune cells, such as T cells and NKs [79,80]. Moreover, NKT cells have been found to play a role in various immune-related diseases and conditions, including infectious diseases, cancer, and autoimmune disorders [82,83,84,85]. Their functional plasticity and ability to modulate immune responses render them a promising target for immunotherapy approaches [84].

Some other cell types, with various mechanisms, have been described as capable of regulating immune responses.

In this regard, the anti-inflammatory role of regulatory B cells (Bregs) has been described [86]. Bregs represent a subset of B lymphocytes with immunosuppressive functions, mainly mediated by the production of anti-inflammatory cytokines such as IL-10, IL-35, and TGF-β [86,87]. Bregs are characterized by differential expression of CD5 and CD1d in the mouse immune system and CD24 and CD38 in the human immune system [86,87,88]. Some evidence suggests that Bregs are involved in infections, inflammation, and autoimmunity [86].

NK cells [1,2,3,4,5,12,13,14,89] are a vital component of the innate immune system, and although their primary role is to recognize and eliminate infected or abnormal cells, a large body of literature suggests that they also play a role in immune regulation [90,91,92]. NK cells recognize and kill tissue cells that display abnormal characteristics, such as infected cells, tumor cells, or cells lacking major histocompatibility complex class I (MHC-I) molecules, based on the missing-self hypothesis [93]. NK cytotoxic function helps prevent the spread of infections and the development of tumors [89,93]. NK cells can also produce numerous cytokines that have both pro-inflammatory and immunosuppressive effects, thus contributing to immune regulation [90,91,92,93]. In addition, they also contribute to immune tolerance by shedding potentially harmful autoreactive or infected cells and sparing healthy ones [94,95,96,97]. NK cells and Tregs can interact, influencing the balance between the activation and inhibition of immune responses [98,99]. Furthermore, NK cells play a crucial role in establishing immune tolerance during pregnancy, facilitating the development of a semi-allogeneic fetus (with different genetic material) within the maternal environment and preventing its rejection [92].

Gamma delta (γδ) T cells are a subset of T lymphocytes that possess a T cell receptor (TCR) composed of γ and δ chains, in contrast to the more common α and β chains of conventional T cells [100,101]. γδ T cells are a relatively small population of T cells in the peripheral blood and have more limited diversity than αβ-TCRs, which allows them to recognize a distinct set of antigens, including non-peptide molecules [101,102]. γδ T cells are often found in tissues such as the skin, the intestinal mucosa, and the respiratory epithelium [102]. γδ T cells contribute to immune surveillance by recognizing and responding to a wide range of stress-induced or non-peptide antigens, such as those produced by infected or transformed cells [101,102]. They can also produce numerous cytokines, such as IFN-γ and TNF-α, which influence the immune response [101]. For their production and roles, they have been implicated in some autoimmune diseases, where they can contribute to inflammation and tissue damage [101].

Dendritic cells (DCs) and macrophages are key players in the immune system, and their versatility extends beyond their role as immune sentinels and scavengers and their known ability to present antigens to T lymphocytes [1,2,3,4,5,6,7,8,9,10]. Indeed, DCs and macrophages are specialized antigen-presenting cells (APCs) that have attracted attention for their intriguing immunomodulatory properties, which allow them to fine-tune immune responses based on the unique signals they encounter and the specific context of the immune challenge [1,2,3,4,5,6,7,8,9,10]. DCs can also interact with Tregs and other immune modulators to further optimize the immune response [103]. Therefore, DCs serve as central coordinators in the immune response, ensuring the body’s defenses are alert against threats (immunogenic DCs) and the immune responses are contained to prevent damage to one’s own tissues (tolerogenic DCs) [104]. This immunomodulation testifies to the complexity of our immune system and its ability to maintain balance in the face of different challenges. Macrophages can assume distinct functional states based on the signals they receive. They can be “classically activated” (M1) to promote inflammation and defense against pathogens or “alternatively activated” (M2) to resolve inflammation, promote tissue repair, and suppress excessive immune responses, reflecting their ability to influence immune modulation [105,106].

Myeloid-derived suppressor cells (MDSCs) represent a heterogeneous group of leukocytes with the ability to suppress immune responses [107,108]. MDSCs originate from myeloid progenitor cells [107,108]. Under certain pathological conditions, such as chronic inflammation or cancer, MDSCs can undergo expansion and become an important component of the immune cell population [107,108].

Finally, the literature highlights a pathogenetic role for some clusters of circulating cells (CIC cells) [109]. CICs express different genetic markers (see previous reference), and there is evidence that the loss of function of specific CIC populations is a contributing factor in T1D [109,110].

## 3. A New Cell Candidate for Immune Regulation: The T_R3-56_

In 2020, we investigated the role of CD3+CD56+ regulatory T cells in the progression of type 1 diabetes (T1D) [111]. We found that individuals with T1D had a significant reduction in the number of CD3+CD56+ regulatory T cells compared to healthy individuals [111]. Such an occurrence was associated with an increase in the activation and effector functions of CD8+ T cells, which are known to contribute to the destruction of insulin-producing beta cells in the pancreas [111]. The study also demonstrated that the reduced numbers of CD3+CD56+ regulatory T cells correlated with disease progression in T1D patients. The decline in these regulatory T cells was associated with increased insulin requirements, indicating a worsening of the disease [111]. Overall, the study suggested that the loss of CD3+CD56+ regulatory T cells contributes to the progression of T1D by allowing for the activation and effector functions of CD8+ T cells. The findings highlight the importance of these regulatory T cells in maintaining immune tolerance and controlling autoimmune responses in T1D.

In this study, we also demonstrated that this CD3+CD56+ T regulatory subset [111] is different from the NKT subset [78,79,80,81,82,83,84,85]. Specifically, CD3+CD56+ regulatory cells (i) are not CD1d-restricted; (ii) do not express Valpha24/Vbeta11 chains but display a heterogeneous V-beta repertoire; and (iii) are unable to kill K562 cells in vitro. In addition, (iv) only 1–5% of CD1d-restricted T cells are positive for the CD56 molecule. We also demonstrated that this CD3+CD56+ regulatory subset is genetically, metabolically, and functionally distinct from the NKT subset [111].

We called this subset T_R3-56_ [111].

In addition, we investigated the role of bone marrow T_R3-56_ cells in patients with very-low-risk/low-risk myelodysplastic syndrome (MDS) [112,113] according to the Revised International Prognostic Scoring System (IPSS-R) [112,113]. MDS comprises a group of blood disorders characterized by ineffective hematopoiesis and a consistent risk of leukemia evolution [112]. We found that in patients with very-low-risk/low-risk MDS, there was an inverse association between the number of T_R3-56_ cells and the activation and expansion of bone marrow cytotoxic T cells [112,113]. Such evidence suggests that T_R3-56_ cells may play a role in regulating the activity of cytotoxic T cells in the bone marrow. Furthermore, the study showed that T_R3-56_ cells from MDS patients exhibited a regulatory phenotype and were capable of suppressing the proliferation and activation of cytotoxic T cells [112,113]. This indicates that T_R3-56_ cells may have immunosuppressive functions in the bone marrow microenvironment, as we previously described for Tregs [114]. Indeed, the imbalance between T_R3-56_ cells and cytotoxic T cells in the bone marrow of very-low-risk/low-risk MDS patients may contribute to the immune-mediated elimination of healthy hematopoiesis, affecting MDS pathogenesis. On the other hand, an increased number and activity of T_R3-56_ cells could contribute to the generation of an immune-suppressed microenvironment in high-risk MDS, which may contribute to the progression of acute leukemia [112,113].

Moreover, we also described the role of T_R3-56_ in chronic lymphocytic leukemia (CLL) with stable disease [115]. We observed that the Treg and T_R3-56_ percentages decreased when evaluated in the context of total lymphocytes. However, when specifically analyzed in the T cell compartment alone, the Treg and T_R3-56_ percentages decreased in CLL subjects. Furthermore, the absolute number of circulating Treg and T_R3-56_ cells is significantly higher in CLL patients than in healthy controls. Since lymphocytes are mainly composed of B cells in CLL patients, the small percentage of T cells within the lymphocyte compartment appears to exhibit a preferential expansion of the Treg and T_R3-56_ regulatory cell subsets as a possible immune escape mechanism [115].

**Figure 1:**
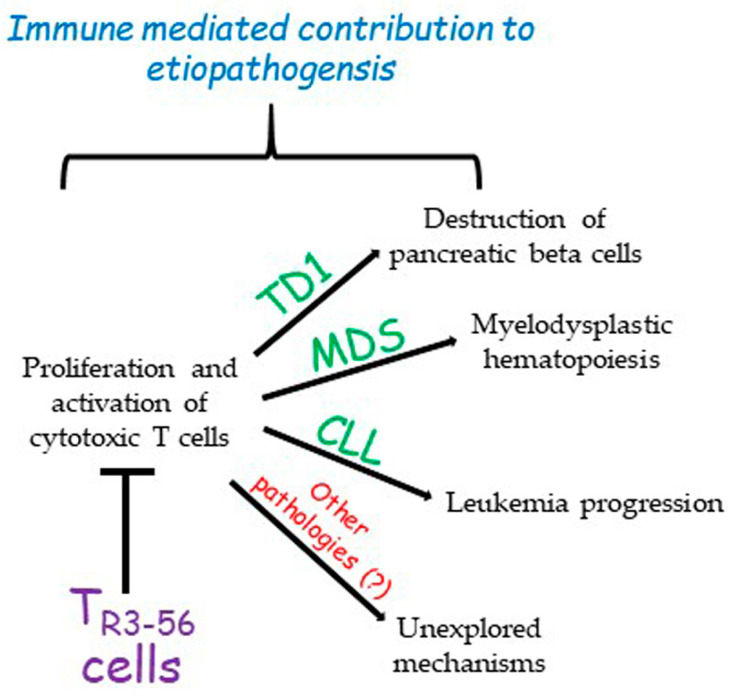
The described role of T_R3-56_ cells. So far, T_R3-56_ cells have been described as playing a role in the determinism of type 1 diabetes (TD1), myelodysplastic syndromes (MDSs), and chronic lymphocytic leukemia (CLL). However, it is possible that this regulatory cell population could be involved in other pathologies. This contribution remains to be explored.

The role of T_R3-56_ cells in the regulation of immune response in specific contexts such as diabetes, cancer, or MDS opens a new scenario towards the possibility of individuating possible molecular targets on these cells to tune the control that this cell subset exerts over the immune system (Figure 1).

For instance, diabetes represents a typical disease for which an effective therapy has not been precisely identified, considering that insulin administration per se does not preserve organs and tissues from the pathological consequences of a hyperglycemic environment [116,117,118]. Indeed, focusing on the role of T_R3-56_ cells in their modulatory action over CD8+ cytotoxic lymphocytes could represent a favorable target to keep the self-destruction of pancreatic cells releasing insulin under control. The identification of specific targets/pathways on these cells could lead to the generation of monoclonal antibodies or small synthetic molecules able to intervene in the treatment of diabetes, better controlling the disease progression and allowing for second-organ preservation. Similarly, this approach could be pursued in the fields of cancer and MDS.

## 4. Previous Observations on CD3+CD56+ Co-Expressing T Cells in Cancer Immune Surveillance

Several studies in recent decades have detected a T lymphocyte population co-expressing CD3+CD56+ molecules, often defining it as NKT-like cells, giving a confusing and non-definitive characterization of the phenotype and role of these cells. CD3+CD56+ T cells are increased in the peripheral blood of patients with solid tumors [119,120]. Such immune cells have been observed in women undergoing in vitro fertilization treatments [121]. A role for CD3+CD56+ T cells has been reported in the pathogenesis of non-alcoholic fatty liver disease [122] and in the development of allergic and autoimmune disorders [123]. Several studies have evaluated the contribution of the CD3+CD56+ T cell population in the pathophysiology and evolution of hematological malignancies: CD3+CD56+ T cell dysfunction has been hypothesized to contribute to the failure of the host immune response against leukemic blasts in acute myeloid and acute lymphocytic leukemia patients [124]; CD3+CD56+ T cells are expanded in the bone marrow of patients with chronic myeloid leukemia (CML) [125] and are decreased in CML patients treated with tyrosine kinase inhibitors [126]; and a higher proportion of CD3+CD56+ lymphocytes has been revealed in lymph nodes affected by large B cell lymphoma [127].

Overall, all these data reveal a general increase in the number of CD3+CD56+ T lymphocytes in cancer patients without addressing a possible explanation for this phenomenon.

Therefore, it is legitimate to argue that the current knowledge does not allow a definitive understanding of these cells. However, a more extensive phenotypic and functional characterization of all the lymphocyte subtypes co-expressing CD3 and CD56 represents the only approach to determining their role and possible involvement in effector and/or immune regulation mechanisms. In this regard, our original and pioneering research on T_R3-56_ cells in the TD1, MDS, and CLL models revealed the phenotypic and functional characteristics of this distinct subpopulation of CD3+ CD56+ T cells, highlighting its distinctiveness in immunoregulation [111,112,113,114].

Nonetheless, it is currently not possible to exclude that CD3+CD56+ cell phenotypes are more numerous or that plastic elements may influence their functions.

## 5. Conclusions

**Figure 2:**
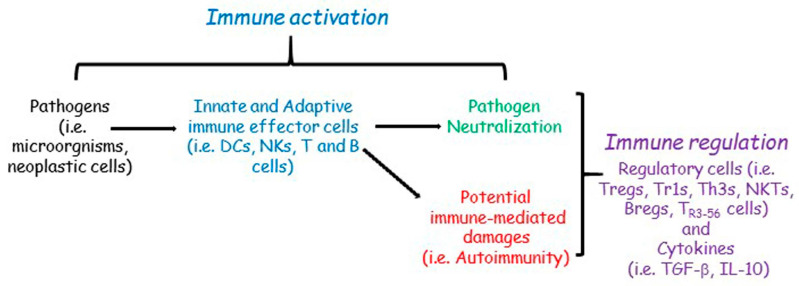
Simplified immune plasticity network. Pathogens activate the innate and adaptive immune effector cells (dendritic cells, DCs; natural killers, NKs; T and B cells) that induce pathogen neutralization during the immune activation phase. However, immune activation could also exert potential immune-mediated damage as a sort of side effect. The immune regulation phase (T regulatory cells, Tregs; Type 1 regulatory cells, Tr1s; T helper 3 cells, Th3s; natural killer T cells, NKTs; B regulatory cells, Bregs; T CD3+ CD56+ regulatory cells, T_R3-56_ cells; transforming growth factor beta, TGF-β; interleukin 10, IL-10) modulates immune activation and avoids immune-mediated damages.

The immune response is ultimately the result of a balance between activation and inhibition; the success and/or failure of the immune response depends on a set of genetic/epigenetic factors and an array of molecules, cells, and tissue microenvironments involved in both activating and inhibitory mechanisms of immune regulation (Figure 2).

The knowledge of all the mechanisms involved in immune regulation is not yet definitive, and, probably, the overall picture is much broader than what has been described in the scientific literature so far. Given the plasticity of the immune system and the diversity of organisms, it is highly probable that numerous other cells and molecules are still to be ascribed to the immune regulation process.

Therefore, it cannot be excluded that other factors and cells other than those reported in this review should be taken into consideration to fully understand the complex harmony between the activation and inhibition of the immune system.

At the same time, it is equally probable that some current knowledge about the role of cells that have hitherto been specifically described as immunoregulatory might need to be revised. Such cells might have different, broader, and more plastic roles in the complex balance between the activation and inhibition of innate and adaptive immune responses.

In this complex framework, it appears highly compelling to propose to the scientific community the investigation of some “new cells”, such as T_R3-56_, in their role as immunoregulatory cell populations, contributing to deepening our knowledge of the immune system and its plastic and dynamic complexity.
